# Cross-sectional study of the association between asthma and cataract among 40 years and older in the USA

**DOI:** 10.1186/s12886-022-02564-y

**Published:** 2022-08-10

**Authors:** Wenwei Li, Bin Wang

**Affiliations:** Department of Ophthalmology, Tongde Hospital of Zhejiang Province, 234 Gucui Road, Hangzhou, 310012 China

**Keywords:** Asthma, Cataract, Epidemiology

## Abstract

**Background:**

This study is aimed to assess the association between asthma and cataract in a representative sample in the United States.

**Methods:**

The National Health Interview Survey (NHIS) is the principal source of information on the health of the civilian noninstitutionalized population of the United States. Ten years (2010-2019) of NHIS were analyzed in this study. Asthma and cataract status were collected from relevant questionnaires among participants aged 40 years and older. Multivariate regression analyses were applied to explore the association between asthma and cataract.

**Results:**

From 40,457 participants included, those with asthma had higher prevalence of cataract than those without asthma (29.41% vs 25.87%, *p* < 0.001). Participants who had asthma had 40% higher odds of cataract compared to those without, after adjusting for potential confounding covariates (Odds Ratio [OR] = 1.40, 95% Confidence Interval [CI]: 1.29-1.52, *p* < 0.001). When viewing asthma as the outcome, participants who had cataract had 36% higher odds of asthma compared to those without, after adjusting for potential confounding covariates (Odds Ratio [OR] = 1.36, 95% Confidence Interval [CI]: 1.25-1.47, *p* < 0.001).

**Conclusions:**

With our study, we demonstrated that individuals with asthma were more likely to develop cataract compared with those without asthma. Further RCTs are needed to confirm this potential impact of asthma on cataract and to investigate the underlying mechanisms.

## Background

As the leading causes of preventable blindness and main causes of visual impairment globally, cataract is deemed to be a principal public health problem in an estimated 52.6 million people [1]. Along with the rapidly aging population, it becomes a huge burden for the developing countries as well as the developed countries. Risk factors such as aging, smoking, alcohol drinking, hypertension, diabetes mellitus, have contributed to the development of cataract [2–6]. Currently, the roles that asthma play in the development of cataract have drawn public attention [7, 8].

As a common chronic inflammatory disease, asthma involves a lot of cells and cellular components in respiratory system. The most common symptoms include shortness of breath, cough, insomnia, fatigue, loss of concentration, wheezing and chest tightness. In some severe case, it may even cause death. It is predicted that about 262 million people suffered from asthma in 2019 and 461,000 deaths are related to asthma, and the number is still on the rise [9].

The National Health Interview Survey (NHIS) is an annual cross-sectional household survey dating back to 1963, which is designed to get a representative sample of US households residing in the United States. It collects data about demographic, socioeconomic and health information from a representative sample of US households residing in the United States. As far as we are aware, there is no definite relation reported between asthma and cataract. Therefore, we conducted this cross-sectional study to investigate the relationships between cataract and asthma using the data from NHIS, 2010–2019.

## Materials and methods

### Sample and population

**Fig. 1:**
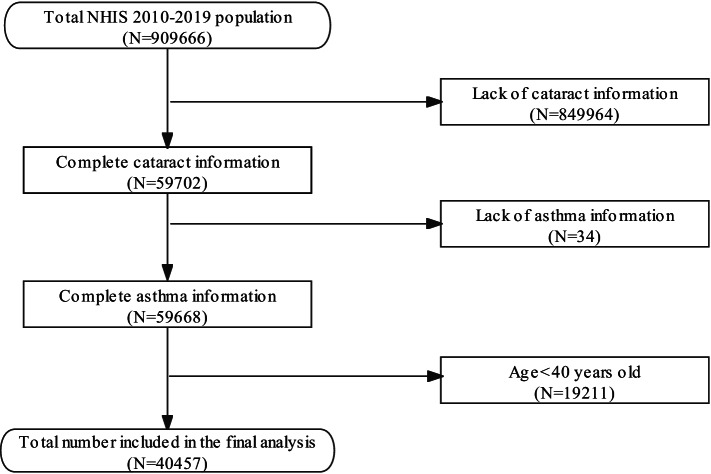
Flowchart for National Center for Health Statistics (NCHS) 2010–2019 about the association between asthma and cataract

The NHIS is designed and operated by the National Center for Health Statistics (NCHS) [10]. Health-related interviews, examinations, and nutrition surveys were collected for late analysis in the NHIS. NHIS data is publicly available and de-identified data, therefore our study was approved by the National Center for Health Statistics research ethics review board. (http://www.cdc.gov/nchs/nhis/about_nhis.htm) Our study collected data from 2010 to 2019 year sample. As shown in Fig. 1, the number of total participants identified in our study is 909,666. According to the survey in NHIS, participants who were 18 years or older were investigated by the question “Ever told have cataracts”. Participants who were younger than 18 years old were not included. Besides, owing to the nature of the survey, some participants did not respond to the question. Due to these reasons, 849,964 participants were excluded with missing information on the cataract. Besides, by reason of missing information on the asthma, 34 participants were excluded. In addition, since most studies involving cataract took participants who were 40 years or older as study’s subject [11–13], 19211 participants were excluded on account of the participants age younger than 40 years old in our study. Finally, 40,457 participants were included in the final analysis.

### Main exposures and outcome variables

Diagnosed with asthma was our main exposure. Asthma was assessed according to the question “Ever told had asthma?” with answers of “Yes” or “No”. Cataract was our primary outcome. Cataract was judged in accord with the question “Ever told have cataracts” with answers of “Yes” or “No”.

### Definition of the covariates

Age, sex, ethnicity, education level, marital status, high cholesterol, diabetes, hypertension, smoking status, alcohol consumption, were esteemed as potential covariates. Ethnicity was classified as non-hispanic and hispanic. Education level was classified as less than or equal to high school degree and more than high school diploma. Marital status was divided as married/living with a partner, and widowed/divorced/separated/never married. High cholesterol, diabetes, hypertension, were all classified as a two-level covariate (no or yes). Smoking status was classified as two groups: current/former and never. Alcohol consumption was classified as lifetime abstainer, former drinker and current drinker.

### Statistical analysis

Means (±standard deviations) was applied when describing continuous variables. Frequencies and percentages were used to describe categorical variables. Independent samples t test was used to analyze normally distributed continuous variables; Mann–Whitney U test was used to analyze non-normally distributed continuous variables. Student’s t-test and χ2 or Fisher’s exact test was used to make a comparison between two groups. To control for confounding factors and identify independent risk factors for cataract, we used logistic regression. Risk covariates with a univariate association of *P* < 0.05 were eligible for inclusion in the multivariate logistic regression model. The results were presented as odds risk (OR) and 95% confidence interval (CI). *P* < 0.05 was considered statistically significant. R (http://www.Rproject.org) was applied to perform all statistical analysis.

## Results

Characteristics of the included participants according to asthma status are presented in Table 1. Compared with participants without asthma, those who suffered from asthma were inclined to be younger, female, non-hispanic, high educated (more than high school diploma), widowed/divorced/separated/never married, current/ former smoker, former/current drinker. They were also less healthy, with high cholesterol, having diabetes and hypertension. Those with asthma had higher prevalence of cataract than those without asthma. (29.41% vs 25.87%, *p* < 0.001).

**Table 1:** Characteristics of participants according to asthma

Characteristics	No asthma	Asthma	*P*-value
No. of participants	35,237	5220	
Age (years)	61.43 ± 12.60	59.92 ± 11.99	< 0.001
Gender			< 0.001
Male	16,305 (46.27%)	1856 (35.56%)	
Female	18,932 (53.73%)	3364 (64.44%)	
Ethnicity			0.006
Non-hispanic	31,883 (90.48%)	4785 (91.67%)	
Hispanic	3354 (9.52%)	435 (8.33%)	
Education			< 0.001
≤ High school	12,808 (36.49%)	1728 (33.21%)	
> High school	22,293 (63.51%)	3475 (66.79%)	
Marriage status			< 0.001
Married/Living with partner	18,717 (53.23%)	2504 (48.13%)	
Widowed/Divorced/ Separated/Never married	16,446 (46.77%)	2699 (51.87%)	
High cholesterol			< 0.001
No	20,663 (58.83%)	2755 (52.97%)	
Yes	14,458 (41.17%)	2446 (47.03%)	
Diabetes			< 0.001
No	29,124 (82.72%)	3937 (75.45%)	
Yes	6084 (17.28%)	1281 (24.55%)	
Hypertension			< 0.001
No	19,028 (54.07%)	2317 (44.44%)	
Yes	16,162 (45.93%)	2897 (55.56%)	
Smoking status			< 0.001
Current/ former	15,729 (44.81%)	2583 (49.63%)	
Never	19,374 (55.19%)	2621 (50.37%)	
Alcohol consumption			< 0.001
Lifetime abstainer	6157 (17.74%)	803 (15.56%)	
Former drinker	6492 (18.70%)	1180 (22.86%)	
Current drinker	22,066 (63.56%)	3178 (61.58%)	
Cataract			< 0.001
No	26,122 (74.13%)	3685 (70.59%)	
Yes	9115 (25.87%)	1535 (29.41%)	

Characteristics of the included participants in accordance with cataract status are presented in Table 2. Compared with participants without cataract, those who had cataract were inclined to be older, female, non-hispanic, high educated, widowed/divorced/separated/never married, current/ former smoker, former/current drinker. They were also less healthy, with high cholesterol, having diabetes and hypertension. Those with cataract had higher prevalence of asthma than those without cataract. (14.41% vs 12.36%, *p* < 0.001).

**Table 2:** Characteristics of participants according to cataract

Characteristics	No cataract	Cataract	*P*-value
No. of participants	29,807	10,650	
Age (years)	57.27 ± 11.04	72.35 ± 9.36	< 0.001
Gender			< 0.001
Male	14,147 (47.46%)	4014 (37.69%)	
Female	15,660 (52.54%)	6636 (62.31%)	
Ethnicity			< 0.001
Non-hispanic	26,603 (89.25%)	10,065 (94.51%)	
Hispanic	3204 (10.75%)	585 (5.49%)	
Education			< 0.001
≤ High school	10,177 (34.28%)	4359 (41.06%)	
> High school	19,512 (65.72%)	6256 (58.94%)	
Marriage status			< 0.001
Married/Living with partner	16,628 (55.92%)	4593 (43.21%)	
Widowed/Divorced/ Separated/Never married	13,108 (44.08%)	6037 (56.79%)	
High cholesterol			< 0.001
No	18,875 (63.52%)	4543 (42.84%)	
Yes	10,842 (36.48%)	6062 (57.16%)	
Diabetes			< 0.001
No	25,384 (85.22%)	7677 (72.15%)	
Yes	4401 (14.78%)	2964 (27.85%)	
Hypertension			< 0.001
No	17,633 (59.22%)	3712 (34.92%)	
Yes	12,141 (40.78%)	6918 (65.08%)	
Smoking status			< 0.001
Current/ former	13,010 (43.81%)	5302 (49.97%)	
Never	16,687 (56.19%)	5308 (50.03%)	
Alcohol consumption			< 0.001
Lifetime abstainer	4799 (16.36%)	2161 (20.51%)	
Former drinker	4977 (16.96%)	2695 (25.58%)	
Current drinker	19,564 (66.68%)	5680 (53.91%)	
Asthma			< 0.001
No	26,122 (87.64%)	9115 (85.59%)	
Yes	3685 (12.36%)	1535 (14.41%)	

Tables 3 and 4 reveal the relationship between asthma and cataract with multivariable logistic regression models. Viewing cataract as the outcome, participants who suffered from asthma had a significantly higher odds of suffering from cataract in logistic regression models. In the crude model, those with asthma had 19% higher odds of cataract than those without asthma (OR = 1.19, 95% CI 1.12 – 1.27, *p* < 0.001); After controlling for age, sex, ethnicity, education level, marital status, those with asthma had 57% higher odds of cataract than those without asthma (OR = 1.57, 95% CI 1.45 – 1.70, *p* < 0.001); Further adjustment with high cholesterol, diabetes, hypertension, smoking status, alcohol consumption, those with asthma had 40% higher odds of cataract than those without asthma (OR = 1.40, 95%CI: 1.29–1.52, *p* < 0.001).

**Table 3:** Logistic regression models of asthma for cataract status

ASTHMA	Crude Model		Model I		Model II	
	OR (95%CI)	*P*-value	OR (95%CI)	*P*-value	OR (95%CI)	*P*-value
No	Reference		Reference		Reference	
Yes	1.19 (1.12, 1.27)	< 0.001	1.57 (1.45, 1.70)	< 0.001	1.40 (1.29, 1.52)	< 0.001

Crude Model adjust for: none.

Model I adjust for: age, sex, ethnicity, education level, marital status.

Model II adjust for: age, sex, ethnicity, education level, marital status, high cholesterol, diabetes, hypertension, smoking status, alcohol consumption.

*Abbreviations*: *CI* Confidence interval

**Table 4:** Logistic regression models of cataract for asthma status

CATARACT	Crude Model		Model I		Model II	
	OR (95%CI)	*P*-value	OR (95%CI)	*P*-value	OR (95%CI)	*P*-value
No	Reference		Reference		Reference	
Yes	1.19 (1.12, 1.27)	< 0.001	1.51 (1.40, 1.64)	< 0.001	1.36 (1.25, 1.47)	< 0.001

Crude Model adjust for: none

Model I adjust for: age, sex, ethnicity, education level, marital status

Model II adjust for: age, sex, ethnicity, education level, marital status, high cholesterol, diabetes, hypertension, smoking status, alcohol consumption

*Abbreviations*: *CI* Confidence interval

When viewing asthma as the outcome, participants who suffered from cataract had a significantly higher odds of suffering from asthma in logistic regression models. In the crude model, those suffering from cataract had 19% higher odds of asthma than those without cataract (OR = 1.19, 95% CI 1.12 – 1.27, *p* < 0.001); After controlling for age, sex, ethnicity, education level, marital status, those suffering from cataract had 51% higher odds of asthma than those without cataract (OR = 1.51, 95% CI 1.40 – 1.64, *p* < 0.001); Further adjustment with high cholesterol, diabetes, hypertension, smoking status, alcohol consumption, those suffering from cataract had 36% higher odds of asthma than those without cataract (OR = 1.36, 95%CI: 1.25–1.47, *p* < 0.001).

## Discussion

In this pooled analysis of nationally representative samples (40,457 US participants aged 40 years old and older in 10 years), we observed that a positive correlation existed between asthma and cataract after adjusting for confounding factors.

Although literature as to the association between asthma and cataract is seldom reported, there is still debate about the findings of earlier research on the relationship between asthma and cataract. Some studies indicate that asthma may positively impact the development of cataract. What we have found in this study is identical with those who have estimated the relationship between these two diseases [7, 8, 14, 15]. Zhao et al. found that when establishing asthma model of rats, asthmatic rats were more likely to develop monocular or binocular cataract than normal rats. PI3K-AKT-mTOR signaling pathway plays an important role in both diseases, which is demonstrated by many clues. Their study demonstrated that asthma may have a close relationship with cataract through the PI3K-AKT-mTOR signaling pathway, resulting in inflammation or immune imbalance on the basis of allergy leading to cataract. MAPK and NF-κB signaling pathways are also important in the development of asthma and cataract. Rho/Rock signaling pathway, Notch signaling pathway, Wnt/β-catenin signaling pathway, JAK/STAT signaling pathway, TGF-β1/Smad signaling pathway may also be involved in the pathogenesis of the two diseases [7, 8]. Lee et al. found that cataracts were significantly associated with asthma and allergic rhinitis by analyzing Korean National Health and Nutrition Examination Survey data [11]. Nevertheless, Maspero found that there were no clinically relevant trends in the assessment of lenticular change during treatment of asthma patients [15].

The positive association between asthma and cataract presented in our study may be explained with the reasons of pathological and therapeutic factors. Pathologically, PI3K-AKT-mTOR, MAPK and NF-κB signaling pathways play an important role in the development of asthma and cataract. Asthma and cataract interacts with each other by means of theses signaling pathways. Therapeutically, the glucocorticoid used in the asthma may have some effects on the development of cataract, especially posterior subcapsular cataract.

With our study, we found that asthma was a high risk factor for cataract and cataract was a high risk factor for asthma as well. Our study is by far the first study to reveal the association between asthma and cataract after adjusting with known confounders.

Our study has several strengths. NHIS is a nationally representative sample with a giant sample size, which includes both genders from the whole nation. In our study, we adjusted for several covariates which comprise age, sex, ethnicity, education level, marital status, high cholesterol, diabetes, hypertension, smoking status, alcohol consumption. And the time span in our study is 10 years. Nevertheless, our study has several limitations. First of all, since our study was a cross-sectional designed study, causal relationship between asthma and cataract could not be determined from our study. Also, since we can not adjust for all factors potentially confounding the asthma and cataract, residual confounding still exists. On the other hand, since inhaled corticosteroid treatment is always applied in clinic for asthma, corticosteroid is usually related with posterior subcapsular [16–20]. However, the data in our study could not differentiate the types of cataract, which might have a bias towards the result. Besides, the analysis of our study included self-report covariates, which may result in misclassification of outcome and information bias. Self-reported asthma and cataract are prone to bias. In order to rule out the possibility of information bias from self-report, a more objective way should be applied to measure asthma, cataract, and covariates of interest in future studies.

## Conclusions

In conclusion, we found that asthma are associated with higher odds of cataract. In the future, more studies are needed to confirm our findings and illustrate the possible mechanisms underlying the relationship between asthma and cataract.

## Data Availability

The datasets used and/or analyzed during the current study are available from the corresponding author on reasonable request.
